# Psycho-demographic profile in severe asthma and effect of emotional mood disorders and hyperventilation syndrome on quality of life

**DOI:** 10.1186/s40359-020-00498-y

**Published:** 2021-01-06

**Authors:** Lucía Dafauce, David Romero, Carlos Carpio, Paula Barga, Santiago Quirce, Carlos Villasante, María Fe Bravo, Rodolfo Álvarez-Sala

**Affiliations:** 1 Psychiatry and Psychology, Hospital Universitario La Paz, Universidad Autónoma de Madrid, CIBERES, Madrid, Spain; 2 Pneumology, Hospital Universitario La Paz, Universidad Autónoma de Madrid, CIBERES, Madrid, Spain; 3 Allergology Services, Hospital Universitario La Paz, Universidad Autónoma de Madrid, CIBERES, Madrid, Spain

**Keywords:** Asthma, Quality of life, Anxiety, Depression, Alexithymia, Hyperventilation syndrome

## Abstract

**Background:**

Severe asthma affects a small population but carries a high psychopathological risk. Therefore, the psychodemographic profile of these patients is of interest. A substantial prevalence of anxiety, depression, alexithymia and hyperventilation syndrome in severe asthma is known, but contradictory results have been observed. These factors can also affect patients’ quality of life. For this reasons, our purpose is to evaluate the psychodemographic profile of patients with severe asthma and assess the prevalence of anxiety, depression, alexithymia and hyperventilation syndrome and their impact on the quality of life of patients with severe asthma.

**Methods:**

A cross-sectional study of 63 patients with severe asthma. Their psychodemographic profile was evaluated using the Hospital Anxiety and Depression Scale (HADS), Toronto Alexithymia Scale (TAS-20), Nijmegen questionnaire and Asthma Control Test (ACT) to determine the state of anxiety and depression, alexithymia, hyperventilation syndrome and control of asthma, respectively. Quality of life was assessed with the Mini Asthma Quality of Life Questionnaire (Mini-AQLQ).

**Results:**

The mean age was 60 ± 13.6 years. Personal psychopathological histories were found in 65.1% of participants, and 8% reported previous suicidal attempts. The rate of anxiety and/or depression (HADS ≥ 11) was 68.3%. These patients present higher scores on the TAS-20 (p < 0.001) for the level of dyspnea (p = 0.021), and for emotional function (p = 0.017) on the Mini-AQLQ, compared with patients without anxiety or depression. Alexithymia (TAS-20 ≥ 61) was observed in 42.9% of patients; these patients were older (p = 0.037) and had a higher HADS score (p = 0.019) than patients with asthma without alexithymia. On the other hand, patients with hyperventilation syndrome (Nijmegen ≥ 23) scored higher on the HADS (p < 0.05), on the Mini-AQLQ (p = 0.002) and on the TAS-20 (p = 0.044) than the group without hyperventilation syndrome. Quality of life was related to anxiety-depression symptomatology (r =  − 0.302; p = 0.016) and alexithymia (r =  − 0.264; p = 0.036). Finally, the Mini-AQLQ total score was associated with the Nijmegen questionnaire total score (r =  − 0.317; p = 0.011), and the activity limitation domain of the Mini-AQLQ correlated with the ACT total score (r = 0.288; p = 0.022).

**Conclusions:**

The rate of anxiety, depression, alexithymia and hyperventilation syndrome is high in patients with severe asthma. Each of these factors is associated with a poor quality of life**.**

## Background

Asthma is a chronic respiratory disease with a high prevalence and incidence. In Spain, its prevalence is approximately 5% [1–3]. Within the total population of patients with asthma, there is a subgroup of patients diagnosed with severe asthma. In these individuals, symptoms of asthma persist, impacting morbidity despite proper pharmacological treatment with correct adherence [4, 5].

Severe asthma is a multifactorial disorder involving, among others, ambient and psychological factors. The Global Initiative for Asthma defines severe asthma as that which remains uncontrolled despite proper use of the recommended treatment and with maximal optimised adherence [2]. However, the few studies that have examined the psychodemographic domain of those with severe asthma have found high rates of psychomorbidity, suggesting a high risk of psychopathology [6–8]. Strong emotions, stress and periods of anxiety and/or depression can lead to exacerbations in such patients [9, 10].

The anxiety and depression combination has a high prevalence in severe asthma [4, 6] and affects the prognosis of the disease, increasing the probability of complications. It is also associated with greater morbimortality compared with that of individuals without emotional disorders [1, 2, 4, 5]. Consequently, it has been demonstrated that anxiety-depression symptomatology in severe asthma is produced because of the irregularity of asthma attacks, dyspnea, visits to emergency departments, hospital stays and the adverse effects of certain medical treatments [10–12]. Furthermore, patients' perception of having received ineffective treatments to control their asthma can also lead anxiety-depression symptoms [10, 13], and with a reduction in quality of life [14–16]. Furthermore, certain characteristics implicit in the anxiety and depression combination, such as uncertainty, low self-esteem and self-efficacy and negative and catastrophising thoughts influence the patient’s perception of the disease and, consequently, their well-being [14, 15]. However, not all the findings are similar in this regard. Some studies have suggested that there are differences in the extent to which each psychiatric symptom independently impacts the disease [7, 13]. Thus, Lavoie et al. [13] were only able to demonstrate that depression, and not anxiety, was associated with poorer control of asthma. On the other hand, Álvarez et al. [7] found discrepancies in the prevalence of anxiety and depression in a population with severe asthma (68% vs. 31%, respectively). Therefore, although these emotional disorders appear to be strongly associated with severe asthma, it is still not known whether the impact of one is greater than the other on the quality of life of such patients.

Furthermore, anxiety and depression are closely linked to misperception, an implicit concept in the psychological construct of alexithymia [17, 18]. Alexithymia is a nosological entity of great scientific and clinical interest in the field of severe asthma. It can be defined as a neuropsychological disorder, including an inability to discern between emotions and bodily sensations, difficulty expressing emotions, having a lack of imagination or fantasy life and thoughts focused on external experience [19]. Alexithymic individuals with asthma tend to underestimate the importance of asthma symptoms or its discomfort. As a consequence, they postpone rescue and/or maintenance medication, increasing the number of visits to emergency departments and hospital stays [14, 20, 21]. The alexithymic traits in these patients can also impede the quality of effective doctor-patient communication [22], therefore biasing the interpretation of their clinical manifestations and favouring overdiagnosis [23]. It is for this reason that alexithymia has recently been included as a risk factor in some official guides on asthma [1, 3]. Alexithymia has also been associated with poor control of asthma and an increased number of life-threatening exacerbations [20, 21]. However, few studies have explored the effect of alexithymia on the quality of life in asthma [14, 24], and even fewer concerning severe asthma [22, 25]. Moreover, the majority of studies have been conducted on populations with different severities of asthma; have used generic and nonspecific questionnaires to evaluate quality of life; or have included a small number of individuals with severe asthma [22, 25].

Another factor to consider in relation to anxiety and alexithymia is hyperventilation syndrome [14], a condition possibly caused by psychological factors, defined as a pattern of breathing deeper and faster than normal. It can be accompanied by dyspnea, chest pain, paraesthesia symptoms, peripheral tetany, palpitations and vertigo, and it frequently appears with anxiety disorders [26].

Despite the apparent robustness of the relationship between severe asthma and various psychological aspects inherent to the disease and to quality of life, there has been little research evaluating the psychodemographic profile of this disease in detail, and the results have been heterogeneous.

For this reason, the aim of this study was to evaluate the psychodemographic profile, the rate of anxiety, depression, alexithymia and hyperventilation syndrome, and their association with quality of life in patients with severe asthma.

## Methods

A descriptive, cross-sectional and observational study was designed. A total of 63 patients diagnosed with severe asthma were consecutively included in the study.

Patients were recruited from the Severe Asthma Clinic in the Pneumology and Allergology Department of a Tertiary Referral University Hospital in the Community of Madrid from January to April 2014. Of the 160 patients initially selected, 97 were excluded for the following reasons: 28 did not wish to participate, 29 did not meet the criteria for being diagnosed with severe asthma, 30 answered the questionnaire incompletely and 10 had incomplete medical records.

The inclusion criteria were as follows: a diagnosis of severe asthma according to the criteria established by the Spanish Society of Pneumology and Thoracic Surgery (SEPAR) [3] and age ≥ 18 years. Exclusion criteria were as follows: active psychosis, serious cognitive impairment and refusal to participate in the study.

After checking the inclusion and exclusion criteria, the study procedures were explained to the patients. Those patients who agreed to the procedures signed the informed consent. All participants were allowed to withdraw their informed consent at any point during the process. The research was approved by the Clinical Research Ethics Committee (*Comité de Ética de la Investigación con Medicamentos*). The committee’s reference number of the study is PI-2553. This investigation was developed according to the principles of the Declaration of Helsinki and its subsequent amendments [27]. All participants followed the protocol used in the severe asthma clinics.

An interview was conducted to collect psychodemographic data, and the Mini Asthma Quality of Life Questionnaire (Mini-AQLQ), Hospital Anxiety and Depression Scale (HADS), Toronto Alexithymia Scale (TAS-20), Nijmegen questionnaire and Asthma Control Test (ACT) were completed.

The interview was based on the Mini-International Neuropsychiatric Interview (MINI) for the evaluation of the patient’s psychopathology [28, 29]. The first part consisted of questions based on this interview as a screening procedure to exclude active psychosis or serious cognitive impairment [29]. Psychodemographic details were collected in the second part of the interview.

### Psychodemographic profile

The information collected was related to the following aspects:

- Sex, age, civil status, level of studies and profession
- Family and personal psychopathological history
- Social and family support to cope with the disease
- Suicide attempts
- Employment situation: active employment, unemployment, retirement, leave or unable to work
- Exacerbating factors explained by the patient: Patients were asked an open question about the exacerbating factors perceived as causes of asthmatic crisis in the last 2 years, including respiratory infection, allergy and emotional stress (understood as the feeling of physical or emotional strain).

### Quality of life

The Mini-AQLQ questionnaire was used to evaluate quality of life in asthmatic adult patients with asthma. It was developed by Juniper, [30] et al. and validated in Spanish by Sanjuàs et al. [31]. It consists of 15 items with an equidistant 7-point Likert response scale, in which 1 is the maximum limitation and 7 is considered to be an absence of limitation [30, 31].

The questionnaire provides an overall score, which is the mean for all the items, and a score for each dimension, which is the mean of the corresponding items. It is divided into 4 dimensions: symptoms, limitation of activities, emotional function and environmental stimuli [30, 31]. It is a simple, short and easy-to-apply test, and it is specific to asthma [30–32].

### Anxiety and depression

The HADS questionnaire was used to evaluate anxiety and depression [33]. This instrument was developed to detect states of anxiety and depression and to differentiate psychological symptoms from somatic ones. The original version was created by Zigmond and Snaith [33] and its adaptation to Spanish was validated in Mexico by Tejero et al. [34]. It is a screening instrument for patients of nonpsychiatric hospital services and contains 14 questions divided into 2 subscales: anxiety (7 items) and depression (7 items), with scores that range from 0 to 21. The total score (anxiety and depression) ranges from 0 to 42 on a 4-point Likert scale with an interval from 0 to 3, in which 0 is “never” and 3 “virtually all day” [33, 34]. The results make it possible to classify patients as having anxiety and depression if their score is ≥ 11 [33, 34].

### Alexithymia

Alexithymia was evaluated using the TAS-20 scale. The instrument was originally developed by Taylor et al. [35] with 26 items; however, it was Parker et al. [36] who validated it with the 20 items that currently comprise the scale. Martínez-Sánchez [37] adapted it to Spanish. It is a quantitative scale with responses scored on a 5-point Likert scale with a numerical interval from 1 to 6, in which 1 means strong disagreement and 6 strong agreement [36]. The overall result has a response interval from 20 to 120 points, and a score ≥ 61 indicates alexithymia [36, 37].

### Hyperventilation syndrome

The Nijmegen questionnaire is a screening method for the early identification of hyperventilation syndrome [38]. This instrument was translated and validated into Spanish in the population with asthma in 2005 by Martínez-Moragón et al. [39]. Its sensitivity is 91% and its specificity is 95% [39]. It has 16 items and 3 components (dyspnea and central and peripheral tetany). The frequency of sensations and symptoms related to hyperventilation syndrome is evaluated through a response scale ranging from 0 ("never") to 4 ("always"). A score of ≥ 23 is considered hyperventilation syndrome [39]. Numerous articles [38, 39] have demonstrated the effectiveness of this method to obtain a differential diagnosis in patients with chronic respiratory diseases.

### Control of asthma

Asthma control was assessed through the self-administered ACT questionnaire, which assesses asthma symptoms and activity limitations [40, 41]. It was developed by Nathan et al. in 2004 [40] and validated into Spanish in 2007 by Vega et al. [41]. The ACT consists of 5 items with a Likert response scale and a 4-point range. The ACT score ranges from 5 to 25, in which 25 is the maximum score and shows adequate control of the disease. Several studies propose a cutoff point of ≤ 19 to indicate poor disease control [40, 41].

### Statistical analysis

The minimal sample size was calculated for detection, in asthmatic patients, a correlation coefficient of 0.45 between anxiety scores and emotion scores in the Mini-AQLQ [42]. Accepting in alpha risk of 0.05 and a beta risk of 0.1, the sample size required was 39 subjects.

Data were expressed as means (standard deviation) or as number of subjects (percentage), depending on whether they corresponded to quantitative or qualitative variables, respectively. To compare quantitative variables, an analysis of variance or Student’s t-test was used for independent groups and, in the case of qualitative variables, the chi-squared test or Fisher’s test was used. For cases in which normality criteria were not fulfilled, nonparametric tests were used. We used the chi-squared test for quantitative variables and the Mann–Whitney U test and Kruskal–Wallis test for qualitative variables. The correlation between variables was analysed using Pearson’s correlation coefficient. Finally, to investigate variables related to the dependent variable, the quality of life, firstly we categorized this variable in two categories, accordingly to the Mini-AQLQ scores ≤ 3 points (worse quality of life) and ≥ 4 points (better quality of life). After that, an univariate regression was used to test each variable separately, aiming to select those to be integrated into the multiple model. Those with p > 0.20 were selected. The SPSS (IBM SPSS Statistic version 19.0.) programme was used to analyse the data, and differences with p < 0.05 were considered to be statistically significant.

## Results

### Psychodemographic profile

The mean age of the included participants was 60 ± 13.6 years; 53 (84.1%) were women. In terms of civil status, 36 (57.1%) were married or lived with a partner. Furthermore, 2 (3.2%) patients had had no academic studies and 19 (30.2%) were in active employment. Personal psychopathological histories were found in 41 (65.1%) patients, and 34 (54%) patients were observed to have no socio-family support to cope with asthma. Five (8%) patients reported previous suicide attempts (Table 1).

**Table 1:** Characteristics of patients, psychodemographic variables, exacerbating factors and quality of life questionnaire

	Patients (n = 63) ${\overline{{\rm x}}}\quad \sigma$
Age	60 (13.6)
Body Mass Index, kg/m^2^	27.7 (6.2)
Female sex	53 (84.1)
Civil status	
Single	11 (17.5)
Married	36 (57.1)
Separated/divorced	6 (9.5)
Widowed	10 (15.9)
Children	37 (58.7)
Level of studies	
No studies	2 (3.2)
Primary	22 (34.9)
Secondary	21 (3.3)
University	18 (28.6)
Employment situation	
Employed	19 (30.2)
Unemployed	3 (4.7)
Retired	18 (28.6)
Leave or incapacity to work	23 (36.5)
Profession	
Contact with toxic products	26 (41.3)
Working with the general public	30 (47.6)
Other	7 (11.1)
Family psychopathological history	26 (41.3)
Personal psychopathological history	41 (65.1)
No social and family support	34 (54.0)
Previous suicidal attempts	5 (7.9)
Exacerbating factors	
Emotional stress	48 (76.2)
Respiratory infection	34 (54)
Change in environmental conditions	21 (33.3)
Allergy	16 (25.4)
Exercise	9 (14.3)
Menopause	2 (3.2)
Mini-AQLQ total	3.7 (1.3)
Mini-AQLQ symptoms	3.7 (1.5)
Mini-AQLQ limitation of activities	3.7 (1.6)
Mini-AQLQ emotional function	3.9 (1.7)
Mini-AQLQ environmental stimuli	3.6 (1.7)

Data are expressed as the mean (standard deviation) or number (percentage)

${\overline{{\rm x}}}$ mean, *σ* standard deviation, *Mini-AQLQ* Mini Asthma Quality of Life Questionnaire

The patients identified emotional stress (82.7%), respiratory infections (52.6%) and changes in environmental conditions (33.3%) as principal exacerbating factors (Table 1).

### Quality of life

The total mean score in the Mini-AQLQ questionnaire was 3.8 ± 1.4, and the scores in the various dimensions were as follows: symptoms 3.7 ± 1.5; limitation of activities 3.7 ± 1.6; emotional function 3.9 ± 1.7; and environmental stimuli 3.6 ± 1.7. No significant differences were found when comparing the quality of life of the various subgroups after classifying the population according to sex, obesity, civil status, level of studies, employment situation and profession (Table 1).

### Anxiety and depression

The total mean HADS score was 15.3 ± 8.8 (depression subscale: 6.8 ± 4.7; anxiety subscale: 8.2 ± 4.8). It was also observed that 43 (68.3%) asthmatics presented total scores ≥ 11, compatible with the diagnosis of anxiety-depression disorder (Table 2).

**Table 2:** Comparative analysis according to the score obtained in the Hospital Anxiety and Depression Scale questionnaire

	HADS ≥ 11 (n = 43)	HADS < 11 (n = 20)	p
Age	60.7 (13.5)	58.5 (14.3)	0.621
Body Mass Index, kg/m^2^	28.0 (6.5)	27.0 (5.4)	0.807
Female sex	35 (81.4)	18 (90.0)	0.481
Civil status			0.067
Single	4 (36.4)	7 (36.6)	
Married	24 (66.7)	12 (33.3)	
Separated/divorced	6 (100)	0 (0)	
Widowed	6 (60)	4 (40)	
Children	1.5	1.1	0.317
Level of studies			0.414
No studies	11 (52.4)	10 (47.6)	
Primary	8 (36.4)	14 (63.6)	
Secondary	1 (50)	1 (50)	
University	14 (77.8)	4 (22.2)	
Employment situation			0.138
Employed	9 (47.4)	10 (52.6)	
Unemployed	3 (100)	0 (0)	
Retired	14 (77.8)	4 (22.2)	
Leave or incapacity to work	14 (60.9)	9 (39.1)	
Profession			0.202
Contact with toxic products	18 (69.2)	8 (30.8)	
Working with the general public	16 (53.3)	14 (46.7)	
Other	6 (85.7)	1 (14.3)	
Family psychopathological history	19 (44.2)	7 (35.0)	0.587
Personal psychopathological history	32 (74.4)	9 (45.0)	0.045
Social and family support	23 (53.5)	11 (55.0)	1.000
Previous suicidal attempts	5 (11.6)	0 (0.0)	0.149
Mini-AQLQ total	3.6 (1.5)	4.1 (1.1)	0.217
Mini-AQLQ symptoms	3.46 (1.5)	4.12 (1.5)	0.119
Mini-AQLQ limitation of activities	3.7 (1.8)	3.81 (1.1)	0.791
Mini-AQLQ emotional function	3.6 (1.8)	4.5 (1.4)	0.017
Mini-AQLQ environmental stimuli	3.5 (1.7)	3.9 (1.8)	0.415
TAS-20	63.7 (16.6)	43.1 (15.9)	< 0.001
Dyspnea Nijmegen	24.1 (9.8)	20.2 (13.8)	0.021
ACT	13.4 (5.6)	14.5 (5.7)	0.459

Data are expressed as the mean (standard deviation) or number (percentage)

*ACT* asthma control test, *HADS* Hospital Anxiety and Depression Scale, *Mini-AQLQ* Mini Asthma Quality of Life Questionnaire, *TAS-20* 20-item Toronto Alexithymia Scale

When comparing the groups with scores ≥ 11 vs. < 11, significant differences were found in the presence of personal psychopathological histories (74.4% *vs*. 45%; p = 0.045), in the TAS-20 score (63.7 ± 16.6 vs. 43.1 ± 15.9; p < 0.001) and in the level of dyspnea (24.1 ± 9.8 *vs*. 20.2 ± 13.8; p = 0.021) (Table 2).

### Alexithymia

Some 42.9% of the patients with asthma had a score compatible with alexithymia on the TAS-20 scale. The total mean score obtained on the TAS-20 scale was 57.2 ± 18.8, indicating that the group of patients had a total score close to the cut off point for an alexithymia diagnosis (≥ 61 points). When comparing the group of patients with ≥ 61 vs. < 61 points on the questionnaire, significant differences were found in age (61.9 ± 13.1 vs. 58.7 ± 14.1; p = 0.037), civil status (p = 0.024) and the HADS questionnaire score (17.7 ± 7.9 vs. 13.5 ± 9.2; p = 0.019) (Table 3).

**Table 3:** Comparative analysis according to the score obtained in the Toronto Alexithymia Scale

	TAS-20 ≥ 61 (n = 27)	TAS-20 < 61 (n = 36)	p
Age	61.89 (13.1)	58.6 (14.1)	0.037
Body Mass Index, kg/m^2^	27.0 (5.6)	28.2 (6.6)	0.518
Female sex	22 (81.5)	31 (86.1)	0.733
Civil status			0.024
Single	1 (9.1)	10 (90.9)	
Married	17 (47.2)	19 (52.8)	
Separated/divorced	5 (83.3)	1 (16.7)	
Widowed	4 (40)	6 (60)	
Children	1.1 (1.1)	1.5 (1.7)	0.303
Level of studies			0.591
No studies	2 (100)	0 (0)	
Primary	9 (40.9)	13 (59.1)	
Secondary	9 (42.9)	12 (57.1)	
University	9 (50)	9 (50)	
Employment situation			0.802
Employed	7 (36.8)	12 (63.2)	
Unemployed	10 (43.5)	13 (56.5)	
Retired	8 (44.4)	10 (55.6)	
Leave or incapacity to work	2 (66.7)	1 (33.3)	
Profession			0.673
Contact with toxic products	10 (38.5)	16 (61.5)	
Working with the general public	13 (43.3)	17 (56.7)	
Other	4 (57.1)	3 (42.9)	
Family psychopathological history	10 (37.0)	16 (44.4)	0.612
Personal psychopathological history	16 (59.3)	25 (69.4)	0.434
Social and family support	15 (55.6)	19 (52.8)	1.000
Previous suicidal attempts	3 (11.1)	2 (5.6)	0.643
Mini-AQLQ total	4.4 (1.0)	4.35 (1.0)	0.441
Mini-AQLQ symptoms	3.4 (1.5)	3.9 (1.5)	0.275
Mini-AQLQ limitation of activities	3.5 (1.7)	3.9 (1.5)	0.607
Mini-AQLQ emotional function	3.6 (1.8)	4.1 (1.6)	0.254
Mini-AQLQ environmental stimuli	3.5 (1.6)	3.7 (1.8)	0.824
HADS	17.7 (7.9)	13.5 (9.2)	0.019
Dyspnea Nijmegen	28.7 (3.0)	25.6 (4.0)	0.178
ACT	13.6 (5.1)	15.9 (6.0)	0.906

Data are expressed as the mean (standard deviation) or number (percentage)

*ACT* asthma control test, *HADS* Hospital Anxiety and Depression Scale, *Mini-AQLQ* Mini Asthma Quality of Life Questionnaire, *TAS-20* 20-item Toronto Alexithymia Scale

### Hyperventilation syndrome

The mean score on the Nijmegen questionnaire was 22.9 ± 11.2. Furthermore, 29 (34%) patients had a score ≥ 23 on the questionnaire, which indicated hyperventilation syndrome. When comparing those with ≥ 23 vs. < 23 points, it was observed that patients with hyperventilation syndrome had a higher total score on the HADS scale (p < 0.001) and on the anxiety subscale (p = 0.002); as well as in the total score on the Mini-AQLQ (p = 0.002) and in the dimensions of symptoms (p = 0.009), limitation of activities (p = 0.027), emotional function (p = 0.003) and environmental stimuli (p = 0.001). Furthermore, this group of patients recorded a higher score on the TAS-20 scale (p = 0.044) than the group without hyperventilation syndrome. In addition, significant differences were observed in civil status (Table 4).

**Table 4:** Comparative analysis according to the score obtained in the Nijmegen questionnaire

	Nijmegen ≥ 23 (n = 29)	Nijmegen < 23 (n = 34)	p
Age	60.7 (14.4)	59.2 (12.9)	0.562
Body Mass Index, kg/m^2^	28.3 (6.2)	27.2 (6.2)	0.553
Female sex	26 (49.1)	27 (50.9)	0.319
Civil status			0.037
Single	2 (18.2)	9 (81.8)	
Married	19 (52.8)	17 (47.2)	
Separated/divorced	5 (83.3)	1 (16.7)	
Widowed	3 (30)	7 (70)	
Children	1.3 (1.8)	1.3 (1.1)	0.500
Level of studies			0.207
No studies	1 (50)	1 (50)	
Primary	10 (45.5)	12 (54.5)	
Secondary	13 (61.9)	8 (38.1)	
University	5 (27.8)	13 (72.2)	
Employment situation			0.401
Employed	9 (47.4)	10 (52.6)	
Unemployed	0 (0)	3 (100)	
Retired	8 (44.4)	10 (55.6)	
Leave or incapacity to work	12 (52.2)	11 (47.8)	
Profession			0.285
Contact with toxic products	15 (57.7)	11 (42.3)	
Working with the general public	11 (36.7)	19 (63.3)	
Other	3 (42.9)	4 (57.1)	
Family psychopathological history	15 (57.7)	11 (42.3)	0.120
Personal psychopathological history	20 (48.8)	21 (51.2)	0.550
Social and family support	15 (44.1)	19 (55.9)	0.741
Previous suicidal attempts	1 (3.4)	4 (11.8)	0.363
Mini-AQLQ total	3.2 (1.5)	4.3 (1.0)	0.002
Mini-AQLQ symptoms	3.2 (1.6)	4.2 (1.3)	0.009
Mini-AQLQ limitation of activities	3.3 (1.8)	4.1 (1.2)	0.027
Mini-AQLQ emotional function	3.2 (1.8)	4.6 (1.3)	0.003
Mini-AQLQ environmental stimuli	2.8 (1.5)	4.2 (1.7)	0.001
TAS-20	18.7 (8.7)	12.4 (8.1)	0.002
HADS	61.9 (14.5)	53.1 (21.4)	0.044
ACT	13.6 (5.3)	13.9 (5.9)	0.841

Data are expressed as the mean (standard deviation) or number (percentage)

*ACT* asthma control test, *HADS* Hospital Anxiety and Depression Scale, *Mini-AQLQ* Mini Asthma Quality of Life Questionnaire, *TAS-20* 20-item Toronto Alexithymia Scale

### Association between quality of life, anxiety, depression, alexithymia, hyperventilation syndrome and control of asthma

**Fig. 1:**
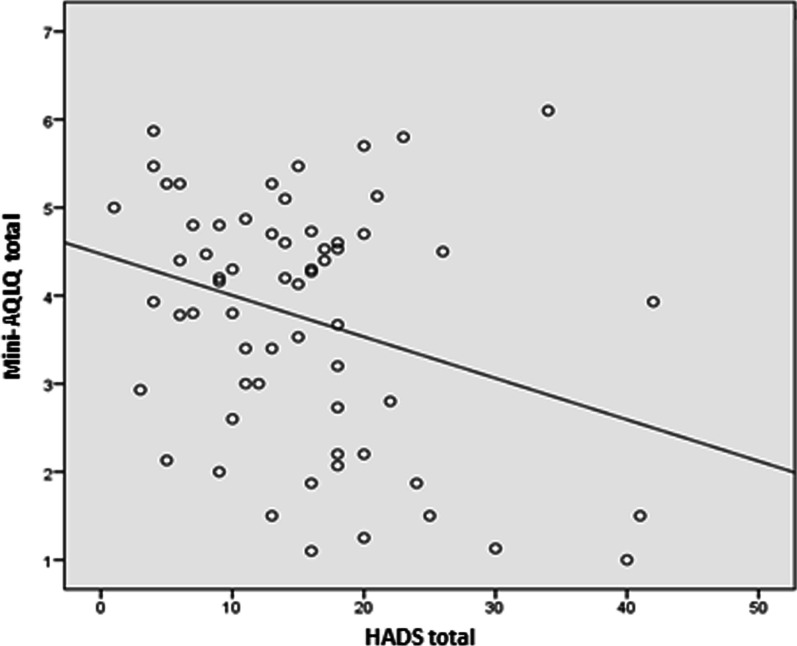
Correlation between anxiety and depression, as evaluated with the Hospital Anxiety and Depression Scale and quality of life as evaluated with the Mini-Asthma Quality of Life Questionnaire. *HADS* Hospital Anxiety and Depression Scale, *Mini-AQLQ* Reduced version of the Asthma Quality of Life Questionnaire

The total score on the HADS scale was significantly related to quality of life (r =  − 0.302; p = 0.016) (Table 5 and Fig. 1). Furthermore, it was associated with the symptoms (r =  − 0.284; p = 0.024) and emotional function (r = -0.358; p = 0.004) dimensions of the Mini-AQLQ questionnaire. Quality of life also correlated with the subscales of anxiety (r =  − 0.317; p = 0.011) and depression (r =  − 0.267; p = 0.034) on the HADS questionnaire (Table 5).

**Table 5:** Correlations between anxiety, depression, alexithymia and quality of life

	HADS		TAS-20		Nijmegen		ACT	
	Correlation	p	Correlation	p	Correlation	p	Correlation	p
Mini-AQLQ total	− 0.302	0.016	− 0.264	0.036	− 0.317	0.011	0.237	0.61
Mini-AQLQ symptoms	− 0.284	0.024	− 0.299	0.017	− 0.312	0.013	0.203	0.110
Mini-AQLQ limitation of activity	− 0.206	0.105	− 0.153	0.230	− 0.154	0.229	0.288	0.022
Mini-AQLQ emotional function	− 0.358	0.004	− 0.305	0.015	− 0.327	0.009	0.72	0.573
Mini-AQLQ environmental stimuli	− 0.147	0.251	− 0.106	0.410	− 0.309	0.014	0.225	0.76

*ACT* asthma control test, *HADS* Hospital Anxiety and Depression Scale, *Mini-AQLQ* Reduced version of the Asthma

Quality of Life Questionnaire; TAS-20: Toronto Alexithymia Scale

**Fig. 2:**
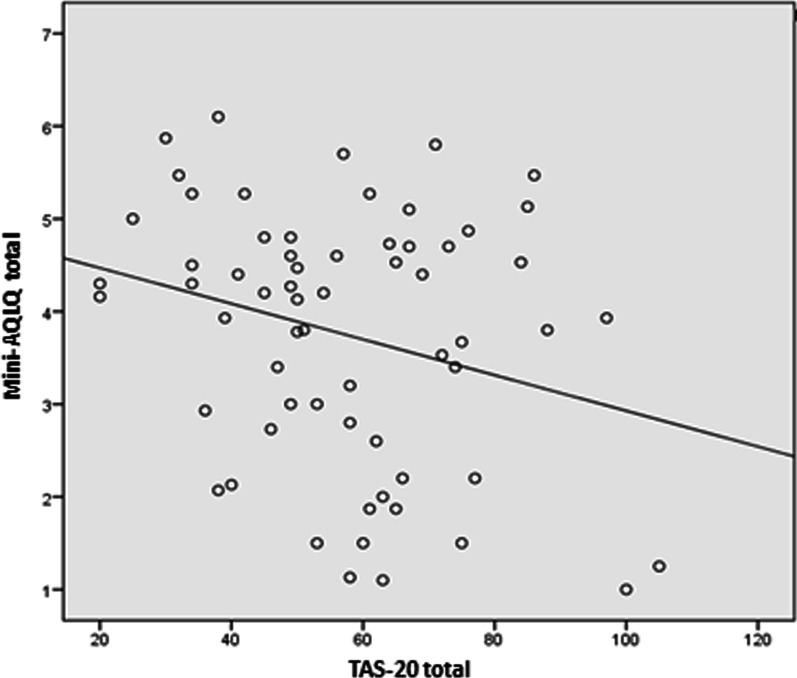
Correlation between alexithymia, as evaluated with the Toronto Alexithymia Scale, and quality of life as evaluated with the Mini- Asthma Quality of Life Questionnaire. *Mini-AQLQ* Reduced version of the Asthma Quality of Life Questionnaire, *TAS-20* Toronto Alexithymia Scale

Furthermore, alexithymia correlated with a poorer quality of life (r =  − 0.264; p = 0.036) (Table 5 and Fig. 2) and with the symptoms (r =  − 0.299; p = 0.017) and emotional function (r =  − 0.305; p = 0.015) dimensions of the Mini-AQLQ. The total score for quality of life was related to hyperventilation syndrome (r =  − 0.317; p = 0.011), as measured with the Nijmegen questionnaire, as well as the subscales symptoms (r =  − 0.312; p = 0.013), emotional function (r =  − 0.327; p = 0.009) and environmental stimuli (r =  − 0.309; p = 0.014) (Table 5). Finally, the limitation of activities of the Mini-AQLQ correlated with the score on the ACT questionnaire (r = 0.288; p = 0.022). In the logistic regression model, hyperventilation syndrome (p = 0.012), TAS-20 scores (p = 0.043), sub-scales of anxiety scores (p = 0.077) an ACT scores (p = 0.091) were selected for the multivariate model. Hyperventilation syndrome was found to be independently associated with the quality of life (OR = 3.973; p = 0.010).

In the logistic regression model, hyperventilation syndrome (p = 0.012), TAS-20 scores (p = 0.043), subscales of anxiety and depression scores (p = 0.077) and ACT scores (p = 0.091) were selected for the multivariate model. Hyperventilation syndrome was found to be independently associated with quality of life (odds ratio 0.252; p = 0.010).

## Discussion

Our study corroborates high rates, close to 60% and 40%, of anxiety-depression symptomatology and alexithymia, respectively. Similarly, it reveals that almost one-third of patients with severe asthma present an associated hyperventilation syndrome. Finally, the anxiety-depression combination, alexithymia and hyperventilation syndrome are associated with a poorer quality of life in these patients.

Asthma impacts negatively on the quality of life of a high percentage of patients [11]. Factors considered associated with a poorer quality of life include being female, smoking, and having a history of poorly controlled or severe asthma, anxiety-depression symptomatology and a sedentary lifestyle [11, 13, 43]. Quality of life in asthma is evaluated using specific scales, such as the AQLQ-Juniper Questionnaire, which has been used in numerous studies and was designed specifically for patients with asthma, [13, 44]. However, one disadvantage of this questionnaire is its length, given it takes up to 10 min to complete [45]. For this reason, smaller and self-administered questionnaires have been designed, which save time both for those who administer them and for patients who complete them [45]. Although the Mini-AQLQ contains only 15 items, it manages to evaluate various dimensions; its strengths are that it is short and self-administered. Given some of our patients had difficulties completing longer questionnaires, we selected the most simplified assessment instruments possible. The results we obtained with the Mini-AQLQ were in line with the literature [42, 46, 47]. Some studies have found that anxiety and depression, as evaluated with the HADS scale, are related to quality of life as measured using the Mini-AQLQ questionnaire, both overall and with its various dimensions [42, 46]. Adeyeye et al. [47] found significant associations between anxiety and depression and the emotional function dimension in an asthmatic population. Our results agreed with these authors in revealing that the anxiety and depression combination is related to the emotional function dimension of the Mini-AQLQ. However, Adeyeye et al. [47] did not find such associations when they evaluated the symptoms dimension of the Mini-AQLQ in patients with severe asthma. Our findings showed that in patients with severe asthma, emotional disorders also have a negative impact on respiratory symptoms in middle-aged patients. Previous studies have analysed this association in older populations and also found it to be significant [46]. However, there is a need for further studies that explore this relationship in severe asthma, given the limited number of studies performed on this type of asthma. It would also be useful to include specific and self-administered questionnaires to evaluate quality of life, because these are the ones that are used in standard practice. Finally, it is necessary to include patients with a wide age range because, from what we have observed, age could be a key factor in the anxiety-depression symptomatology and quality of life association.

With regard to the association between alexithymia and quality of life, we found that patients with alexithymia presented a poorer quality of life than those who did not have it. Several studies that have evaluated this relationship have been performed on small samples of patients with various severities of asthma and have used generic questionnaires to evaluate quality of life [22, 24, 25]. Few studies have evaluated this association in severe asthma, and their results have been similar to ours [22, 25]. Furthermore, only Chugg et al. [22] used the same questionnaires we used in our research, showing that 44% of their patients with asthma fulfilled criteria of alexithymia, a rate similar to that found by us. In their population, the mean scores on the TAS-20 questionnaire were somewhat lower than ours (48.3 vs. 57.2, respectively), undoubtedly related to the lesser degree of severity of the respiratory disease and the smaller sample size. In addition, Vázquez et al. [25], after using a generic evaluation questionnaire on quality of life (health questionnaire SF-36), observed that the alexithymia dimension and the difficulty in identifying feelings were related to the physical function obtained from the SF-36 questionnaire, which is similar to the link we demonstrated between alexithymia and symptoms in the Mini-AQLQ. This similarity could be explained by the difficulty implicit in alexithymia of interpreting emotions not manifested with a somatic representation [48]. Furthermore, the association we found between alexithymia and emotional function on the Mini-AQLQ was similar to the one found by Axelsson et al. [24]. These authors noted a significant relationship between alexithymia and the mental component of the SF-8 questionnaire to measure quality of life, and that this finding negatively impacted on the control of the disease. However, their population was not composed of patients with severe asthma. Furthermore, another study also detected that alexithymia impacts on quality of life as measured using the AQLQ-Sydney questionnaire of patients with poorly-controlled asthma [14]. Consequently, we deduced that alexithymia acts upon the emotions and the symptoms of the disease and that this could be a risk factor in the severity of the disease, in anxiety and depression comorbidity and in the impact on quality of life. However, more studies that explore alexithymia in severe asthma and that measure its impact on various dimensions of the patients’ quality of life are required.

Psychomorbidity in asthma has been highlighted in several studies [4–7, 49]. Recent research suggests that comorbidity between asthma and affective disorders could be due in part to genetic factors shared between asthma and depression [50]. The prevalence of anxiety in this population is notably higher than in the general population (20–27% vs. 4–10%, respectively). The same occurs with depression (17–29% vs. 5–11%, respectively) and for anxious-depressive symptoms only, the prevalence increases to 45% [1–3, 5]. Thus, the more severe the disease, the greater the levels of anxiety-depression symptomatology manifest themselves [51]. However, studies performed on patients with severe asthma are limited; among these, the Analysis of psychological factors in patients with severe asthma study [7] and the study performed by Sastre et al. [6] are remarkable. Both describe a prevalence of anxiety and depression in the population with asthma close to 70%. Our results coincide with those of these authors [6, 7], given 68.3% of our patients displayed anxiety-depression symptoms. Furthermore, like Sastre et al. [6], we used the HADS scale to evaluate the patients’ emotional state. Here, the mean score found was 14.3, somewhat higher than the one described by Sastre et al. (12.4 points) [6]. The difference between these scores can be explained by the fact that the population of Sastre et al. [6] was composed primarily of patients with moderate asthma (81%) and of a younger age than ours (46.8 vs. 60 years, respectively).

Finally, we observed that 54% of our patients did not receive any socio-family support for their disease, which some studies have related to anxiety-depression symptoms and also with poorer control of the disease [5, 8].

Emotional disorders, especially depression, can in the long term manifest themselves as suicide attempts. In our series, 8% of patients had made one or more attempt. Chung et al. [52] and Clarke et al. [53] had detected a rate of 1% and 4.2%, respectively. The lower rate of suicide attempts observed in their populations could be explained by the higher rates of anxiety-depression symptoms presented in our patients (68.3%) compared with those collected by Chung et al. [52] (12%) and Clarke et al. [53] (29.6%). This difference, in turn, could be related to the greater severity of asthma that characterised our study. In addition, they suggest as key factors in the asthma-suicide attempts association, low socioeconomic level, lower level of education, unemployment, limited socio-family support, civil status (divorced/separated or widowed) and/or unhealthy habits (smoking, alcohol consumption and lack of exercise) [52].

The prevalence rate of alexithymia in the general population is between 5 and 10% [14, 21]. In contrast, in the population with asthma this rate increases considerably (38–51%) [14, 54]. Our data reveal that 42.9% of patients with severe asthma present alexithymia. A similar figure was obtained by Innamorati et al. [54], who associated alexithymia with poorer dynamic respiratory functions and with more depressive symptoms. Therefore, alexithymia is not only related to a greater respiratory component, but it could also be associated with a greater severity of the disease. Serrano et al. [20] found that patients with near-fatal asthma scored more than double in alexithymia in comparison with those with a lower severity of the disease. Plaza et al. [21] evaluated alexithymia in a group of asthma patients with a life-threatening history and observed an average score on the questionnaire (TAS-26) somewhat higher than what we observed. These authors had administered the scale that preceded the TAS-20 and the TAS-26, and in a population of limited size. In addition, alexithymia appears to frequently be associated with depressive states and older age. Depression and alexithymia typically appear together. However, they need to be differentiated. This differentiation is complex, given that the symptomology can be similar or because symptoms overlap [48]. They also share a similar neurobiological substrate in which the anterior cingulate cortex, the amygdala and the insular cortex play a key role [48].

These neuroanatomical structures are related to affective flattening, perceptive distortions, emotional regulation and empathy. Similarly, old age can increase the characteristics of the alexithymic personality, given the cognitive deterioration inherent to the natural evolution of humans. As we age, cognitive ability declines and consequently generates a reduction in expressive ability and abstract thinking [48].

We observed that 34% of patients presented hyperventilation syndrome. This finding is consistent with the literature that describes a prevalence of hyperventilation syndrome of 5%–10% in the general population and between 29 and 42% of patients with bronchial asthma [14]. Although there have been few studies on this syndrome in severe asthma, the study by Álvarez et al. [7] described a somewhat higher rate in their sample (47.5%) of patients with severe asthma [7]. Hyperventilation syndrome was associated with a higher score on the overall HADS scale and with more anxiety, more alexithymia and a poorer quality of life overall as well as in each dimension of the Mini-AQLQ. These results demonstrate a high rate of hyperventilation syndrome in severe asthma and in the associated psychopathology, and that it is a risk factor for the development of an alexithymic personality and a component related to a poorer quality of life. These data are coherent with those of Martínez-Moragón et al. [39], who recorded a hyperventilation syndrome rate of 36%, and those of Martínez-Rivera et al. [14], with 38% of this syndrome found among their patients with asthma. The mean we obtained from the Nijmegen test was somewhat higher than that one obtained by Martínez-Rivera et al. [14], which is undoubtedly related to the greater severity of asthma and the older age of our patients. Hyperventilation syndrome occurs in approximately one-third of people with asthma, which could be due to its similarity with the signs of dyspnea, and it is more frequent in severe asthma [5]. It is also associated with greater psychopathology, anxiety, number of exacerbations and a poorer quality of life [5].

Chronic respiratory diseases, such as asthma, result in the development of personal and psychosocial characteristics with a high psychopathological risk, [7–9]. Furthermore, the more serious the underlying pathology, the more likely this risk will increase [4, 7, 9, 49]. Our results support these findings, given we observed that a high percentage of patients with severe asthma had family psychopathological histories and personal psychiatric histories. Recently, Behmanesh et al. [55] found family histories of anxiety, depression and/or stress in more than half of the patients with asthma included in their research.

Along the same lines, some years ago, Wamboldt et al. [56] recorded a high prevalence of psychiatric disorders in the mothers of adolescents with severe asthma and suggested a link between severe asthma, affective disorders and dysfunctional family dynamics. Furthermore, with regard to personal psychiatric histories, Potoczek et al. [57] explored a population with severe asthma using a similar evaluation tool, the MINI, and showed a high prevalence of psychopathological personal histories and suggested that such conditions affected the severity of asthma [57]. A number of publications [13, 23] have demonstrated an association between anxiety, depression and poor asthma control. Poor asthma control also affects patients’ quality of life, according to Axelsson et al. [24]. In our study, we measured asthma control with the ACT, a tool that is widely studied [6, 7, 24] and used in clinical practice, and we obtained significance with the Mini AQLQ symptom scale. These results are similar to those of Axelsson et al. [24], who found that the ACT was a predictor of the physical component of their quality of life questionnaire.

Our study is characterised by high levels of unemployment, primarily due to retirement, work-related disability or sick leave. These results could be influenced by the advanced age of the population, which is inherent to severe asthma. These findings could also be influenced by the high rate of anxiety-depression symptoms observed. Campbell et al. [58] revealed that 40.3% of patients with asthma suffered more than 1 severe exacerbation every week that resulted in an absence from school or work. Also, SEPAR [4, 5] positioned bronchial asthma as the fourth largest cause of workplace absenteeism. The proffesional characteristics of some of our participants, who are exposed to irritants and/or allergens or to the stress inherent in employment positions working with the general public, could suggest the high rate of work-related disability. Psychological aspects play a key role as exacerbating factors of severe asthma [9, 10, 49, 59, 60]; a stressful life situation can precipitate an exacerbation [5, 9, 10, 59, 60]. Some years ago, Akçakaya et al. [9] had described emotional factors as triggers of mild asthma and, even more so, of severe asthma. In addition, Jauregui and Tejedor [60] suggested that stressful stimuli were bronchoconstriction agents in bronchial provocation techniques. Finally, the anxiety component of asthma and exposure to stress could act jointly and influence the onset of exacerbations [10, 59, 60].

A key limitation of our study is the absence of a control group with mild asthma. Also, comparing our findings with those of a group with diagnosis of milder severity asthma would be interesting, and this comparison could increase the results’ degree of evidence. Another important limitation of this study is that we did not include the separate score of the 3 subscales that comprise the alexithymia questionnaire, TAS-20. We were only interested in whether patients had alexithymia as commonly as in the works of Baeza et al. [18] or Ponce et al. [44]. These authors had analysed only the total score of the questionnaire, distinguishing only between patients with or without alexithymia, and did not estimate the subscales within it in their results [18, 44]. However, alexithymia is a multifaceted construct, and the TAS-20 typically includes 3 subscales, 1 of which is not substantially correlated with the other 2. The analysis could be performed separately for the 3 dimensions of alexithymia, and this process could have improved the quality of our results. Another limitation is that the sample size is lower compared with those in the studies by Sastre et al. [6] and Ponce et al. [44], and the number of individuals with severe asthma was greater than that included in other series, such as those of Lavoie, et al. [61] and Valença et al. [62]. Finally, the Nijmegen scale is useless in populations that suffer from asthma or anxiety disorders because it includes items that are specific to these conditions. The scores could be elevated simply by high endorsement of these items without generating any information regarding the tendency to hyperventilate in these patients. However, a previous study [39] had noted that anxiety-panic attacks (states of extreme anxiety) and hyperventilation syndrome sometimes coincide in the same patient, but the prevalence of both disorders is discordant in these populations.

Chronic respiratory diseases have an effect on the personality and emotional state of the individuals who experience them. Specifically, patients with severe asthma evidence a sociodemographic profile with personality traits (alexithymia) and specific levels of psychopathology (anxiety and/or depression). This profile can make it difficult to identify asthma symptoms and could overlap with other clinical conditions, such as hyperventilation syndrome, which can influence quality of life. Thus, our results could have clinical utility given they highlight the advantage of collaborating with a psychologist in multidisciplinary units for patients with severe asthma.

## Conclusions

In summary, the present results suggest that populations diagnosed with severe asthma present specific sociodemographic characteristics indicating a high burden of associated psychopathology. We also conclude that anxiety, depression, alexithymia and hyperventilation syndrome are present in a high percentage of patients with severe asthma and that these act as factors associated with a poorer quality of life for these individuals.

## Data Availability

The datasets used and/or analysed during the current study are available from the corresponding author on reasonable request.

## Abbreviations

ACT: Asthma Control Test
HADS: Hospital Anxiety and Depression Scale
Mini-AQLQ: The Mini Asthma Quality of Life Questionnaire
MINI: Mini-International Neuropsychiatric Interview
SEPAR: Spanish Society of Pneumology and Thoracic Surgery
TAS-20: Toronto Alexithymia Scale
